# New Perspective on the Use of α-Bisabolol for Weed Control

**DOI:** 10.1021/acs.jafc.3c08566

**Published:** 2024-03-19

**Authors:** Josyelem Tiburtino Leite Chaves, Geovane da Silva Dias, Marina Mariá Pereira, Ludmila da Silva Bastos, Maria Isabel Almeida Souza, Larissa Fonseca Andrade Vieira, Ana Cardoso Clemente Filha Ferreira de Paula, Cláudia Araújo Marco, Paulo Eduardo Ribeiro Marchiori, Elisa Monteze Bicalho

**Affiliations:** † Laboratório de Crescimento e Desenvolvimento de Plantas, Setor de Fisiologia Vegetal, Universidade Federal de Lavras, Lavras, Minas Gerais CEP 37200-000, Brazil; ‡ Laboratório de Citogenética, Universidade Federal de Lavras, Lavras, Minas Gerais CEP 37200-000, Brazil; § Instituto Federal de Minas Gerais, Bambuí, Minas Gerais CEP 38900-000, Brazil; ∥ Laboratório Interdisciplinar em Produtos Naturais, Centro de Ciências Agrárias e da Terra, Universidade Federal do Cariri, Crato, Ceará CEP 63130-025, Brazil

**Keywords:** *Vanillosmopsis arborea*, α-bisabolol, bioherbicide, oxidative stress, germination, postemergence

## Abstract

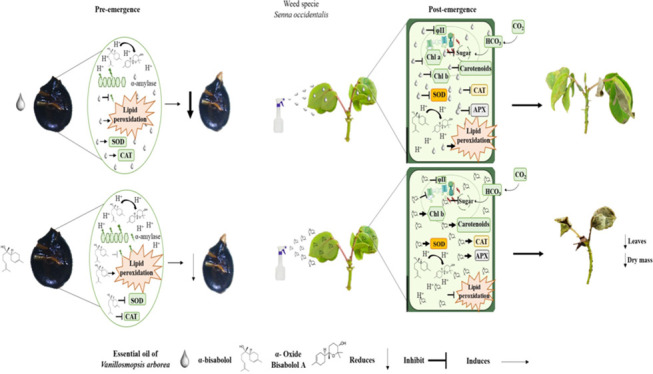

The indiscriminate use of synthetic herbicides reduces its effectiveness. Bioherbicides produced with metabolites emerge as an alternative to managing weeds. We aimed to analyze the phytotoxic potential of the essential oil of *Vanillosmopsis arborea* (EOVA) and the α-bisabolol molecule, its main component. We evaluated the effects of EOVA and α-bisabolol at different concentrations on the germination, growth, antioxidant metabolism, and photosynthesis of different species. EOVA and α-bisabolol showed promising phytotoxic effects on the germination and initial growth of the weed *Senna occidentalis*, inhibiting the activity of the antioxidant enzymes and increasing lipid peroxidation. α-Bisabolol reduced the weed seedling growth by inducing oxidative stress, which suggests a greater role in postemergence. Moreover, in the weed postemergence, both EOVA and α-bisabolol caused damage in the shoots, reduced the chlorophyll content, and increased lipid peroxidation besides reducing photosynthesis in *S. occidentalis*. Overall, we suggest the promising action of α-bisabolol and EOVA as bioherbicides for weed control.

## 1 Introduction

*Vanillosmopsis arborea* Baker (“candeeiro”) (Asteraceae) is an endemic tree of Chapada do Araripe, Crato, CE, Brazil. This species produces an essential oil reported to impair the germination of model species.^1^ Furthermore, this essential oil has high economic and medicinal value, due to its high α-bisabolol content (70–90%).^1^ This molecule is a secondary metabolite of the sesquiterpene type with pharmacological properties such as anti-inflammatory, antibacterial, antifungal, and genotoxic action on glioma tumor cells.^2,3^ Despite the vast literature on α-bisabolol, its influence on plant metabolism remains unknown. Several terpenes have a phytotoxic activity,^4,5^ and the essential oil of *V. arborea* and the α-bisabolol molecule could potentially control weed species.

Terpenes can damage seeds and reduce the rate of plant growth and development. Several allelochemicals alter the redox system of cells by causing an overproduction of reactive oxygen species (ROS), which damages lipids, proteins, and DNA.^6^ Cellular homeostasis is disrupted due to the inhibition of mitochondrial oxygen consumption (respiration) or damage to photosystem II in chloroplast (photosynthesis).^7−9^ Overproduction of ROS and suppression of the activity of antioxidant enzymes cause oxidative stress, decreasing seed germination and plant growth.^10^

Such changes in plant metabolism are particularly interesting for the management of weed species.^4,11^ The indiscriminate use of synthetic herbicides and the lack of products with a specific mode of action favor the emergence of resistant weeds and reduce the effectiveness of these products, increasing production costs and reducing crop productivity.^12^ Thus, bioherbicides produced with metabolites like α-bisabolol emerge as a more environmentally safe and efficient alternative in the management of weeds.^13,14^

Thus, it is hypothesized that the essential oil of *V. arborea* (EOVA) and α-bisabolol (the major component of EOVA) inhibit seed germination and reduce the growth of weed species by inducing oxidative stress and affecting photosynthesis, while in nontarget species (crops), there is no damage. For that, experiments were performed on selected seed-propagating weed species with wide territorial distribution (*Bidens pilosa* L., *Cenchrus echinatus* L., *Cyperus disfformis* L., *Desmodium tortuosum* (Sw.) DC and *Senna occidentalis* L.) and crops (*Lactuca sativa* L. and *Oryza sativa* L.) in the OECD (Organization for Economic Co-operation and Development) guidelines for the testing of chemicals.^14^ Here, we aimed to investigate the phytotoxic potential and action mechanism of the essential oil of *V. arborea* (EOVA) and α-bisabolol (possibly its major component) in the pre- and postemergence of weed and nontarget crop species.

## 2 Material and Methods

### 2.1 Plant Material

*Vanillosmopsis arborea*'s wood was collected from plants located at Chapada do Araripe in Crato-CE, Brazil (7°07′39″S 39°25′32″W). Seeds of five target species were collected from at least 20 plants: *Bidens pilosa* L. (Crato-CE, Brazil 7°14′11″S 39°22′08′′W), *Cenchrus echinatus* L. (Crato-CE 7°14′11″S 39°22′08″W), *Cyperus disfformis* L. (Federal University of Lavras (UFLA) 21°13′36″S 44°58′53′′W), *Desmodium tortuosum* (Sw.) DC (Ijaci-MG, 21°10′08″S 44°54′52′′W) and *Senna occidentalis* L. (Ijaci-MG, 21°10′08″S 44°54′52″W). Seeds of the nontarget species *Lactuca sativa* L. cv. Monica were obtained from an agricultural house in Lavras-MG whereas seeds of *Oryza sativa* L. cv. Caçula (harvested in 2019) were obtained from the Genetics and Plant Breeding Department of UFLA.

### 2.2 Extraction and Analysis of the EOVA

The wood from the lateral branches of three *V. arborea* plants was removed, cut, divided into portions of 500 g, immersed in distilled water, and hydrodistilled in a clevenger-type hydrodistillation apparatus using the methodology of Alencar et al.^15^ The EOVA obtained was stored in an amber bottle at 4 °C. The oil components were identified by gas chromatography coupled to the mass spectrophotometer (GC-MS), model GCMS-QP2010 Ultra (Shimadzu) according to Adams.^16^

### 2.3 Preparation of Solutions with Essential Oil of *V. arborea* and α-Bisabolol

EOVA (100%) or α-bisabolol [sesquiterpene α-bisabolol ((−)-6-methyl-2-(4-methyl-3-cyclohexen-1-yl)-5-hepten-2-ol, Sigma-Aldrich-Merck) was diluted in deionized water at 40 °C for reaching concentrations of 0.125, 0.25, 0.50, 0.75, and 1%. The dilution was performed individually for each experimental replicate, and the solutions’ pH was between 5.5 and 6.2.

### 2.4 Bioassays

The first experiment tested the potential of EOVA to inhibit seed germination and reduce the growth of weed species without damaging nontarget crop species. In the second and third experiments, the mechanisms of action of EOVA and α-bisabolol were compared in the pre and postemergence of seeds from *S. occidentalis* (weed) and *O. sativa* (crop).

#### 2.4.1 Experiment 1: Seed Germination of Target and Nontarget Species under EOVA Application

Seeds of *S. occidentalis* and *C. disfformis* were subjected to a physical dormancy-breaking treatment with sulfuric acid (96%) for 20 and 5 min, respectively, and then rinsed with deionized water three times to remove any traces of the acid.^17^ The seeds of *D. tortuosum* were scarified with sandpaper number 180.^18^

Seeds were disinfected in a solution of 2.5% sodium hypochlorite and detergent for 15 min and then rinsed three times in distilled water before the germination assays. Aliquots of 4 mL of deionized water (control) and each EOVA concentration were used to moisten the filter paper where the seeds were sown in Petri dishes. The entire experiment was carried out in growth chambers set to the optimal temperature and photoperiod for each species to germinate: 25 °C for 24 h in the dark (*B. pilosa* and *L. sativa*), 25 °C and 12 h of light at 40 μmol photons m^–2^ s^–1^ (*O. sativa*), 30 °C and 12 h of light at 40 μmol photons m^–2^ s^–1^ (*C. echinatus*, *D. tortuosum*, and *S. occidentalis* with), and 30 °C (day)/20 °C (night) and 16 h of light at 40 μmol photons m^–2^ s^–1^ (*C. disfformis*).

The experiment lasted until the germination (2 mm protruded radicle) of each target species was completed (i.e., 48 h with no increase in the germination percentage of the control). The number of germinated seeds was recorded every 24 h. The germination percentage (GP) (eq 1) and the germination speed index (GSI) (eq 2) were calculated according to the methods of Ranal et al.^19^ and Maguire,^20^ respectively. The shoot and root sizes of the seedlings were measured (in centimeters) with a graduated ruler. The nongerminated seeds from each treatment were subjected to a viability test with 2–3–5 triphenyltetrazolium chloride solution. Seeds were evaluated by the intensity of the red/pink color in their structures and classified as viable or nonviable (noncolored).
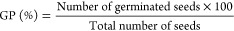
1
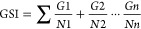
2where *G* = number of germinated seeds in day; *N* = number of day.

#### 2.4.2 Experiment 2: Physiological Effects of EOVA and α-Bisabolol in Seeds and Seedlings of *S. occidentalis* and *O. sativa*

##### 2.4.2.1 Germination Tests

Germination tests were performed under the same conditions described in Experiment 1. Each concentration of EOVA or α-bisabolol was used to moisten the filter paper where the *O. sativa* or *S. occidentalis* seeds were sown.

##### 2.4.2.2 Biochemical Analyses

An imbibition curve was set for both species to determine the timing of seed sampling. During imbibition, five replicates of 25 seeds were weighed until the radicle protrusion. Seeds were sampled during phase II of the germination triphasic pattern (complete imbibition of the seeds),^21^ at 10 (*S. occidentalis*), and 12 (*O. sativa*) hours of imbibition. Seedlings were also sampled at the end of the experiment. Seeds and seedlings were frozen in liquid nitrogen and stored at −80 °C until biochemical analyses.

α-Amylase activity was determined using Miller^22^ method modified by Liu et al.^23^ H_2_O_2_ quantification was performed with 100 mg of seeds or 200 mg of seedlings according to the method of Velikova et al.^24^ The extent of lipid peroxidation was measured by the amount of malondialdehyde (MDA) in 100 mg of seeds according to Du and Bramlage^25^ and 200 mg of seedlings according to Buege and Aust.^26^ For assaying the activity of antioxidant enzymes [catalase (CAT), superoxide dismutase (SOD), and ascorbate peroxidase (APX)], 200 mg of seeds or seedlings was homogenized in phosphate buffer, EDTA, and ascorbic acid.^27^ Proteins were quantified using the Bradford method.^28^ CAT activity was determined by the method of Havir and McHale,^29^ SOD activity as described by Giannopolitis and Ries,^30^ and APX activity according to Nakano and Asada.^31^

#### 2.4.3 Experiment 3: Physiological Effects of EOVA and α-Bisabolol on Postemergence of *S. occidentalis* and *O. sativa*

Seeds were sown in 0.8 L pots containing a mixture of soil (dystrophic Red Latosol) and sand (1:1) and kept in a greenhouse under natural conditions. Twenty days after emergence, two plants per pot were left. Seedlings of *O. sativa* and *S. occidentalis* were sprayed with 4 mL 0.5% EVOA, 0.5% α-bisabolol, or deionized water (control) at 45 (rice) or 120 (weed) days after emergence, which corresponded to the critical period of competition between species.^32^ After applying the treatments, the plants were monitored daily for 5 days (rice) and 40 h (weed) considering the deterioration (leaf necrosis) of the treated plants.

##### 2.4.3.1 Growth Parameters

At the end of the experiment, the number of completely expanded or not expanded leaves was measured as well as the dry matter. Plants were collected, washed in deionized water, separated into roots and shoots, and dried at 60 °C in an oven until a constant weight.

##### 2.4.3.2 Photosynthesis and Chlorophyll Fluorescence

Assimilation of CO_2_ (A) of *O. sativa* was measured from 9 a.m. until 12 p.m. with mean photosynthetically active radiation of 800 μmol m^2^ s^–1^, using a closed system, considering the carbon balance of the entire shoot. A CO_2_ gas analyzer (SBA-5, PP Systems, Amesbury, USA) was used according to the method of Sestak et al.,^33^ as described by Mitchell.^34^ A chamber with a metallic structure covered by a transparent plastic film totaling a volume of 0.0285 m^3^ was built to fit the shoots. Individual plants were placed in the chamber and after stabilization of the initial CO_2_ concentration (400 ± 10 μmol mol^–1^), the decay of CO_2_ concentration (i.e., the net CO_2_ consumption by the plant) was measured for 5 min. The following equation was used to calculate the CO_2_ assimilation in the closed system (eq 1).^34^ Photosynthesis was standardized according to the ambient temperature (30 ± 2 °C), temperature (K), and pressure (Pa).^34^ The leaf area was measured with the Easy Leaf Area app.
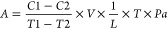
3where *A* = the CO_2_ assimilation (μmol m^–2^ s^–1^); *C*_1_ and *C*_2_ = the CO_2_ (μmol mol^–1^) concentration at times *T*_1_ and *T*_2_; *V* = the total volume of the system (m^3^); *L* = leaf area (m^2^); *T* = temperature (K); and *Pa* is pressure (MPa).

As *S. occidentalis* plants were not large enough to quantify the gas exchange of the entire plant, photosynthesis was determined using a LI-6400XT infrared gas analyzer (LI-COR, Inc., Lincoln, NE, USA) and a leaf cuvette (6400-40, LI-COR, Inc., USA) from 9 a.m. until 12 p.m. Leaf tissue was exposed to 1200 μmol m^–2^ s^–1^ photosynthetically active radiation, a CO_2_ concentration of 400 μmol mol^–1^ at a flow rate of 500 μmol s^–1^, and an ambient temperature of 27 ± 2 °C. The leaf area was measured with ImageJ.

Chlorophyll *a* fluorescence was measured immediately after gas exchange measurements by a MultispeQ spectrophotometer (PhotosynQ) according to Kuhlgert et al.^35^ The RIDES new SPAD DMK photosynthesis protocol was used and is available on the PhotosynQ platform (https://www.photosynq.com/product-page/multispeq-v-2-0).

##### 2.4.3.3 Biochemical Analyses

At the end of the experiment, plants were collected, washed in deionized water, separated into shoots and roots, frozen in liquid nitrogen, and stored at −80 °C. The contents of H_2_O_2_, MDA, and protein as well as CAT, SOD, and APX activity were performed as described above. Photosynthetic pigments were determined with 50 mg of leaf tissue discolored in acetone, and the concentrations of chlorophyll *a*, *b*, and carotenoids were quantified following the methods of Lichtenthaler and Buschman.^36^

### 2.5 Statistical Analysis

Independent experiments for each species were conducted in a completely randomized design. Experiment 1 consisted of six treatments (EOVA concentrations of 0, 0.125, 0.25, 0.50, 0.75, and 1%) with five replicates of 25 seeds each. In Experiment 2, a two-way analysis of variance was performed considering the two potential bioherbicides (EOVA and α-bisabolol) at five concentrations (0, 0.25, 0.50, 0.75, and 1%). Each treatment had five replicates of 25 seeds. Experiment 3 consisted of three treatments (0.5% EOVA, 0.5% α-bisabolol, and deionized water as the control) with seven replicates of four plants each. From each replicate, two plants were used for gas exchange analysis and two for biochemical analyses.

Data were analyzed using the statistical software RBio©^37^ with ExpDes.pt package and subjected to the Shapiro–Wilk normality test and analysis of variance (ANOVA). When significant (*F* test at 5% probability), the data were subjected to regression analysis or Tukey’s test at 5% probability. All equations, *R*^2^ values, and *p* values are shown in the Supporting Information. A multivariate cluster analysis (UPGMA) based on Euclidean distance in percentage was performed considering the GP, GSI, and the length of the shoot and root for all species and concentrations from Experiment 1.

## 3 Results

### 3.1 Effects of the Essential Oil of *Vanillosmopsis arborea* (EOVA) on Pre-Emergence of Target and Nontarget Species

CG-MS analysis of the EOVA identified 11 compounds (Table 1). The major components of the oil were α-bisabolol (93.57%), eugenol (2.14%), bisabolol oxide (1.48%), elemicin (0.67%), and eucalyptol (0.65%).

**Table 1:** Percentage of *Vanillosmopsis arborea* Essential Oil Components Identified by CG-MS

**percentage (%)**	**components**
0.10	3-butenyl propyl ether
0.09	3,3-dimethyl-2-hexanone
0.65	eucalyptol
0.09	terpineol
2.14	eugenol
0.21	*cis*-caryophyllene
0.67	elemicin
0.3	(−)-spathulenol
1.48	oxide bisabolol
93.57	α-bisabolol
0.15	β-chamigrene
0.51	eremanthine
99.96	total

**Figure 1:**
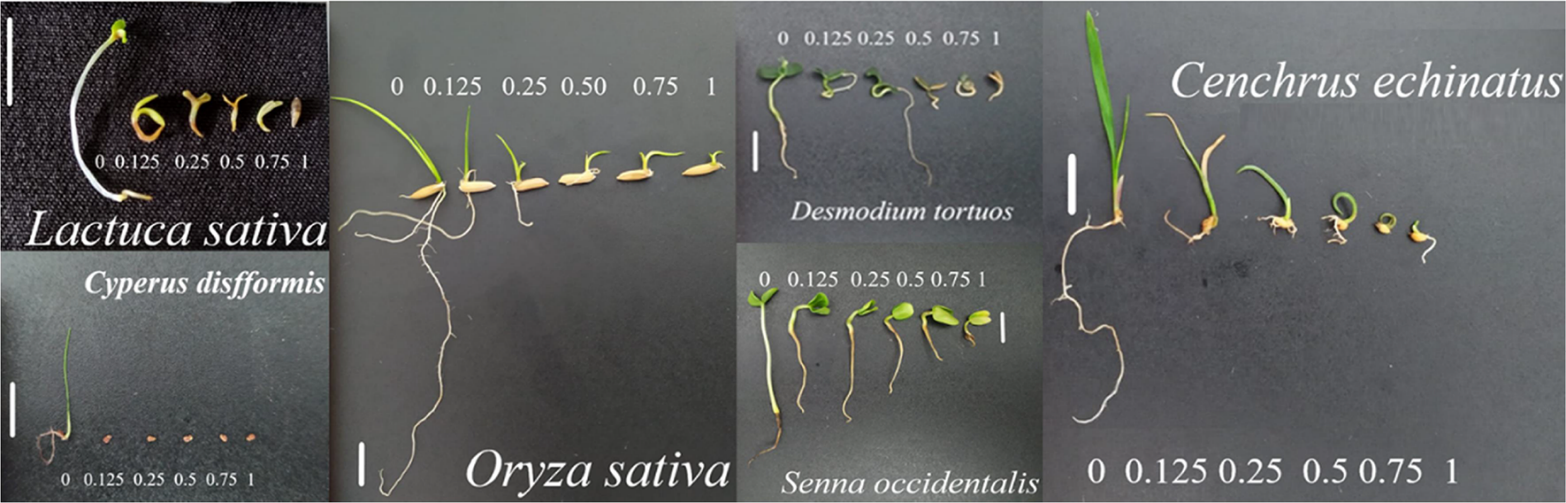
Morphology of seedlings submitted to different concentrations of the essential oil of *Vanillosmopsis arborea*.

EOVA inhibited seed germination of *B. pilosa* but not that of *O. sativa*. EOVA reduced the GP and GSI in a dose-dependent manner in *L. sativa* and *S. occidentalis*. The highest concentration of the EOVA (1%) was the most effective in reducing the GP of *L. sativa*, *S. occidentalis*, and *D. tortuosum*, decreasing the GP by 85, 61, and 27%, respectively. Interestingly, concentrations of 0.125 and 0.25% increased the GSI in *D. tortuosum*. The lowest concentration (0.125%) reduced seed germination in *C. disfformis* and *C. echinatus* by 95 and 87%, respectively, and the GSI was almost null (Table 2). The seedlings’ size of the target and nontarget species was significantly influenced by all treatments (Table 2). The length of the shoot and root of *L. sativa* seedlings was reduced in a dose-dependency manner (Table 2) until total inhibition of seedling development. The root length of *S. occidentalis* (Table 2) was higher at 0.125%. The size of *O. sativa*, *D. tortuosum*, and *C. echinatus* seedlings (Table 2) was reduced in all EOVA concentrations, whereas concentrations of 0.5% onward abolished the growth of roots and shoots in *C. echinatus* (Table 2). Even at the lowest concentrations, EOVA application completely inhibited the growth of *C. disfformis* seedlings (Table 2). Moreover, seedlings of the species *L. sativa*, *O. sativa*, *S. occidentalis*, and *C. echinatus* showed signs of necrosis and chlorosis or impaired growth of roots and shoots at the end of the experiment (Figure 1 and Figure S1). The UPGMA grouped species according to their degree of sensitivity to the EOVA application (Figure S2). Two distinct groups were observed, one including *O. sativa* and *S. occidentalis* and another with the remaining species. The model species *L. sativa* showed medium sensitivity to treatments and was grouped with most weed species. *C. disfformis*, *C. echinatus*, and *B. pilosa* were the most sensitive to the treatments, with the latter showing the highest dissimilarity with *O. sativa*.

**Table 2:** Seed Germination (PG, %), Germination Speed Index (GSI, %), Shoot Length (cm), and Root Length (cm) of Seedlings Submitted to Different Concentrations of the Essential Oil of *Vanillosmopsis arborea*^a^

conc. (%)	*Lactuca sativa*				*Oryza sativa*				*Senna occidentalis*			
	GP (%)	GSI	shoot (cm)	root (cm)	GP (%)	GSI	shoot (cm)	root (cm)	GP (%)	GSI	shoot (cm)	root (cm)
0	98.4 ± 0.86	23.68 ± 0.29	1.22 ± 0.02	0.91 ± 0.02	95.2 ± 1.7	11.29 ± 0.31	3.49 ± 0.17	6.15 ± 0.21	99.2 ± 0.5	22.7 ± 0.82	4.4 ± 0.21	0.62 ± 0.04
0.125	78.4 ± 2.3	14.33 ± 0.86	0.568 ± 0.01	0.53 ± 0.03	93.6 ± 0.8	10.76 ± 0.21	2.08 ± 0.12	2.23 ± 0.15	98.4 ± 0.8	14.56 ± 1.94	1.91 ± 0.09	1.42 ± 0.21
0.25	72.8 ± 2.4	11.81 ± 1.09	0.454 ± 0.01	0.40 ± 0.03	92 ± 1.4	10.14 ± 0.24	1.46 ± 0.03	1.76 ± 0.16	97.6 ± 0.8	16.63 ± 1.53	1.75 ± 0.04	1.13 ± 0.13
0.5	41.6 ± 2.3	6.11 ± 0.76	0.37 ± 0.03	0.30 ± 0.02	93.6 ± 0.8	10.14 ± 0.12	1.32 ± 0.05	1.60 ± 0.14	76 ± 2.1	8.35 ± 0.48	1.18 ± 0.07	1.81 ± 0.12
0.75	36.0 ± 2.1	4.23 ± 0.41	0.354 ± 0.02	0.24 ± 0.02	89.6 ± 0.8	9.29 ± 0.34	1.09 ± 0.15	0.69 ± 0.11	72.8 ± 1.3	7.19 ± 0.68	1.25 ± 0.01	2.17 ± 0.30
1	14.4 ± 0.9	1.55 ± 0.11	0	0	88.8 ± 1.8	9.36 ± 0.22	0.84 ± 0.06	0.54 ± 0.08	38.4 ± 10.1	3.99 ± 1.14	0.8 ± 0.28	2.44 ± 0.38
*R*^2^	0.97	0.96	0.78	0.89	mean = 92.13	0.69	0.87	0.72	0.90	0.82	0.84	0.50

conc. (%)	***Desmodium tortuosum***				***Cenchrus echinatus***				***Cyperus disfformis***			
	PG (%)	GSI	shoot (cm)	root (cm)	PG (%)	GSI	shoot (cm)	root (cm)	PG (%)	GSI	shoot (cm)	root (cm)
0	94.2 ± 1.65	8.6 ± 0.30	1.8 ± 0.06	2.21 ± 0.07	84.8 ± 1.71	12.04 ± 0.55	4.85 ± 0.09	5.65 ± 0.33	74,4 ± 6.93	3.48 ± 0.39	1.64 ± 0.09	0.71 ± 0.03
0.125	91.2 ± 1.86	11.98 ± 0.60	0.80 ± 0.02	1.6 ± 0.03	10.4 ± 4.15	0.58 ± 0.19	0.36 ± 0.19	0.36 ± 0.20	3,2 ± 0.71	0.20 ± 0.07	0	0
0.25	91.93 ± 0.75	12.83 ± 0.21	0.82 ± 0.03	1.70 ± 0.05	12 ± 4.29	0.65 ± 0.22	0.39 ± 0.21	0.39 ± 0.20	4 ± 2.26	0.29 ± 0.02	0	0
0.5	80 ± 2.14	8.53 ± 0.31	0.61 ± 0.05	2.18 ± 0.16	4 ± 0.71	0.2 ± 0.04	0	0	4,8 ± 2.86	0.27 ± 0.02	0	0
0.75	68.56 ± 3.78	7.05 ± 0.51	0.53 ± 0.03	2.30 ± 0.12	3.2 ± 1.14	0.14 ± 0.05	0	0	3,2 ± 1.33	0.25 ± 0.01	0	0
1	68.8 ± 2.43	7.00 ± 0.32	0.37 ± 0.03	2.16 ± 0.13	3.2 ± 1.72	0.06 ± 0.04	0	0	4 ± 1.13	0.27 ± 0.09	0	0
R^2^	0.82	0.68	0.87	0.72	0.93	0.92	0.90	0.91	0.90	0.91	-	-

conc. (%)	***Bidens pilosa***			
	PG (%)	GSI	shoot (cm)	root (cm)
0	91.2 ± 1.45	20.66 ± 0.63	3.58 ± 0.09	2.18 ± 0.07
0.125	0	0	0	0
0.25	0	0	0	0
0.5	0	0	0	0
0.75	0	0	0	0
1	0	0	0	0
R^2^	-	-	-	-

a Data are means ± standard errors (*n* = 5). The regression equations are listed in the Supporting Information Table S1.

### 3.2 Physiological Effects of EOVA and α-Bisabolol on *S. occidentalis* and *O. sativa*

#### 3.2.1 Physiological Responses of Seeds

**Figure 2:**
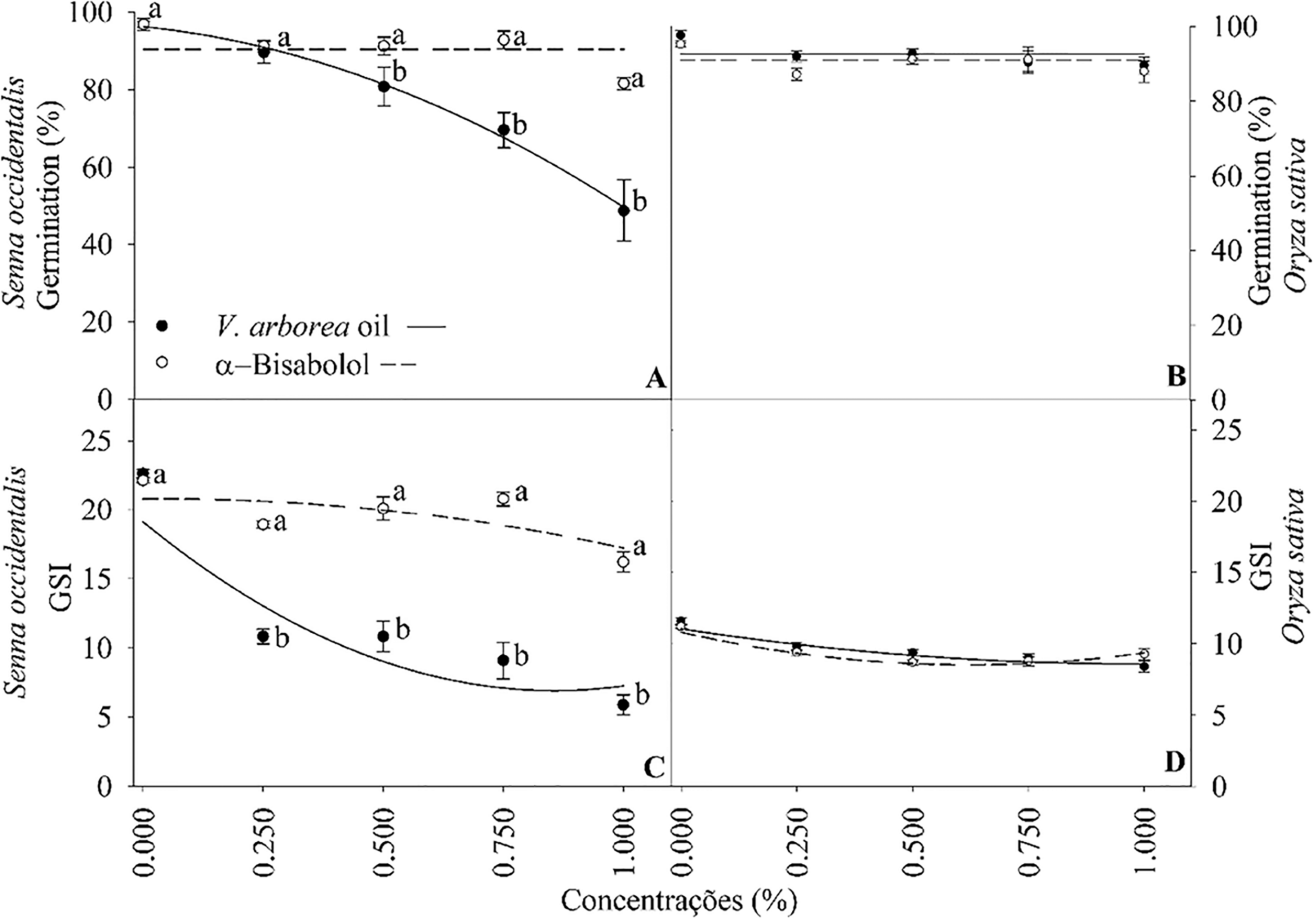
Germination percentage and germination speed index (GSI) of *Senna occidentalis* (A; C) and *Oryza sativa* (B; D) seeds subjected to different concentrations of the essential oil of *Vanillosmopsis arborea* and α-bisabolol. Data are means ± standard errors (*n* = 5). The equations are listed in the Supporting Information Table S2.

As *O. sativa* and *B. pilosa* were the species showing the highest dissimilarity concerning EOVA sensitivity, we focused our efforts on further analyzing their physiological responses. The GP of *S. occidentalis* seeds was significantly affected by the application of EOVA (Figure 2). The highest concentration (1%) reduced GP by approximately 50%. However, α-bisabolol did not have the same effect. The GSI of *S. occidentalis* decreased by approximately 50% in response to the lowest EOVA concentration (0.25%); however, the greatest reduction induced by α-bis(abolol) was 30% at a concentration of 1%. The GP of *O. sativa* seeds was not significantly affected by the treatments (Figure 2), but the GSI was reduced by approximately 30% with EOVA and α-bisabolol at a concentration of 1%.

**Figure 3:**
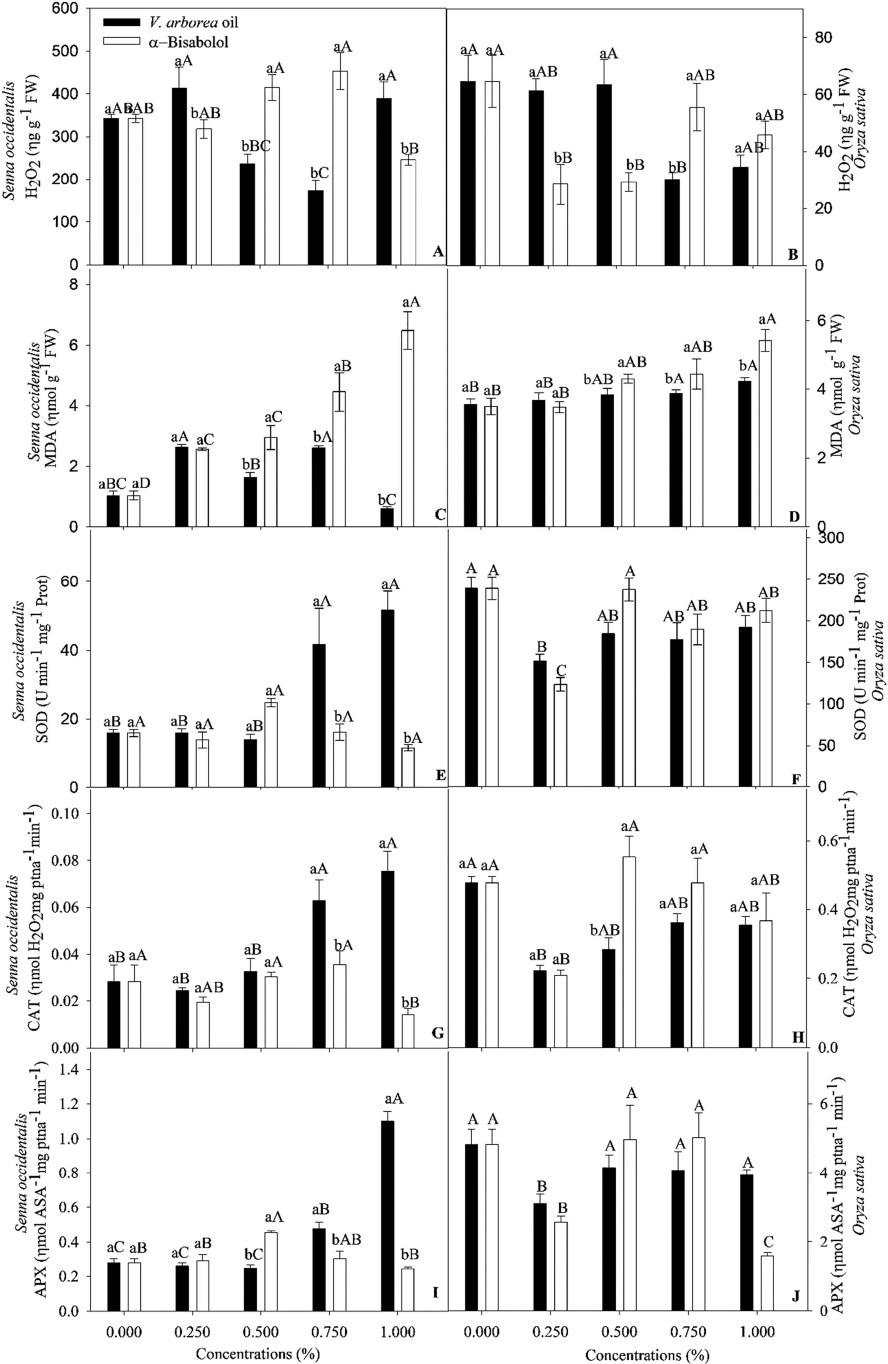
Concentration of H_2_O_2_ and malondialdehyde (MDA) and activity of antioxidant enzymes superoxide dismutase (SOD), ascorbate peroxidase (APX), and catalase (CAT) of *Senna occidentalis* (A; C; E; G; I) and *Oryza sativa* (B; D; F; H; J) seeds subjected to different concentrations of the essential oil of *Vanillosmopsis arborea* and α-bisabolol. Data are means ± standard errors (*n* = 5). Significant differences (*p* < 0.05) are indicated by letters. Lowercase letters compare the essential oil of *V. arborea* and α-bisabolol, and uppercaseletters compare different concentrations.

EOVA at 0.25 and 1% increased the amount of H_2_O_2_ in *S. occidentalis* seeds (Figure 3), but the opposite was observed in response to α-bisabolol application. The levels of malondialdehyde (MDA) also increased by approximately 150% in seeds of *S. occidentalis* treated with 0.25 and 0.75% EOVA (Figure 3), but the MDA concentration was reduced by approximately 40% at 1% EOVA. α-Bisabolol increased the MDA concentration in both species (Figure 3). Regarding antioxidant enzymes, 1% EOVA led to increased activities of SOD, CAT, and APX in *S. occidentalis* seeds (Figure 3). However, this was not the case with α-bisabolol, as only the concentration of 0.5% increased the activity of the three enzymes (Figure 3). The lower EOVA concentrations increased the levels of H_2_O_2_ in *O. sativa* seeds but did not affect the MDA content, whereas α-bisabolol had contrasting results. CAT, APX, and SOD activities decreased in response to both EOVA and α-bisabolol by 0.25% (Figure 3).

#### 3.2.2 Physiological Responses of Seedlings

**Figure 4:**
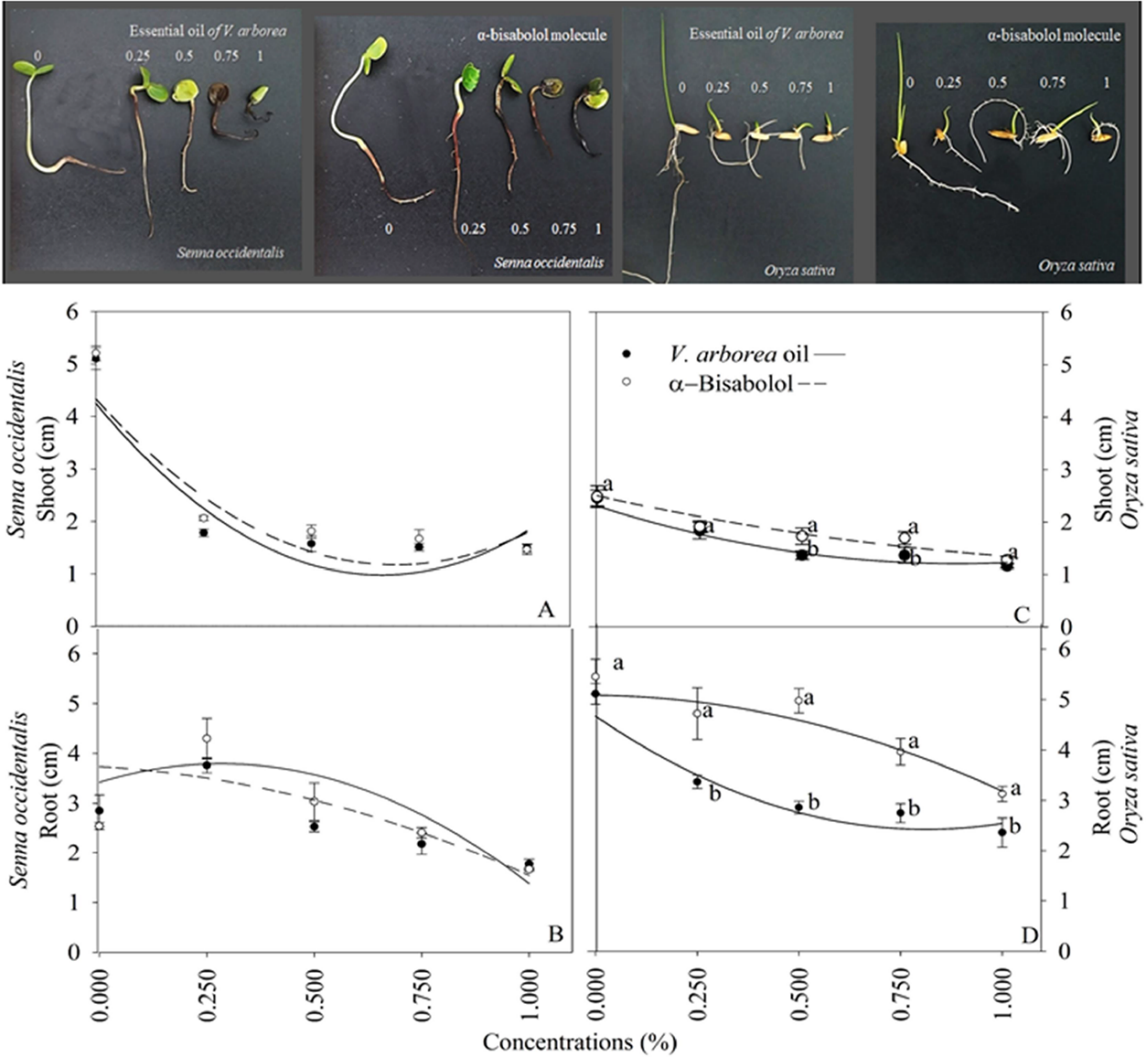
Morphology and length of shoots and roots of seedlings subjected to different concentrations of the essential oil of *Vanillosmopsis arborea* and α-bisabolol. Data are means ± standard errors (*n* = 5). Significant differences (*p* < 0.05) between EOVA and α-bisabolol are indicated by letters The equations are shown in the Supporting Information Table S2.

The shoot and root lengths of *S. occidentalis* seedlings were significantly affected by the treatments (Figure 4). Concentrations of 0.25% strongly decreased the shoot length, while increasing the root length. Seedling root length decreased by approximately 50% with concentrations of 1% of both substances. Seedlings exhibited necrosis when treated with 0.75 and 1% EOVA and with 0.5, 0.75, and 1% α-bisabolol (Figure 4).

The root and shoot length of *O. sativa* seedlings was significantly affected by the treatments (Figure 4) EOVA caused a more drastic reduction in shoot length than α-bisabolol at all concentrations tested. The concentration of 1% of both EOVA and α-bisabolol exerted the greatest reduction in the *O. sativa* shoot length. Compared with α-bisabolol, EOVA caused the largest reduction in the root length of *O. sativa* seedlings with increasing concentrations (Figure 4).

**Figure 5:**
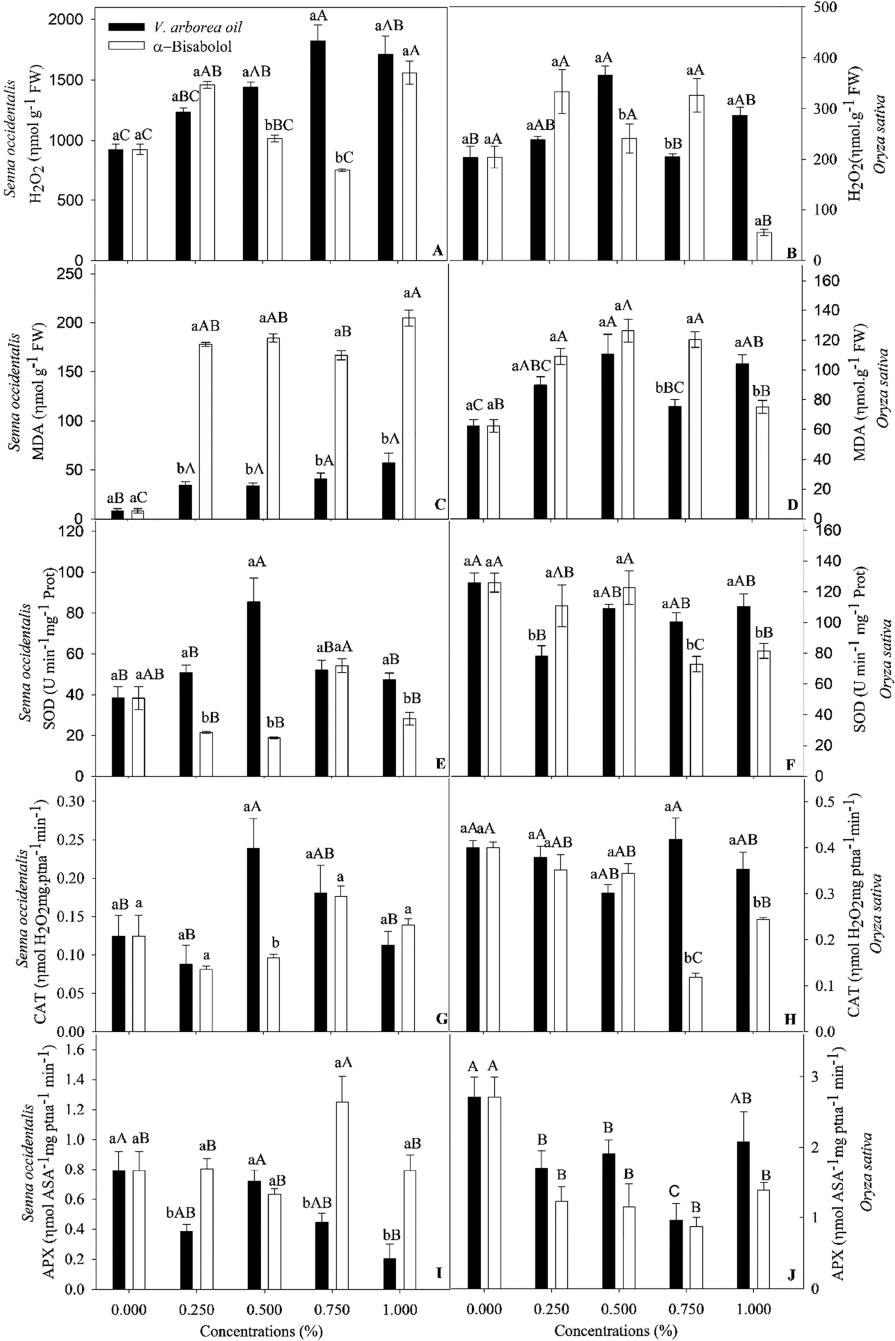
Levels of H_2_O_2_ and malondialdehyde (MDA) and enzyme activity of superoxide dismutase (SOD), ascorbate peroxidase (APX), and catalase (CAT) in *Senna occidentalis* (A; C; E; G; I) and *Oryza sativa* (B; D; F; H; J) seedlings subjected to different concentrations of the essential oil of *Vanillosmopsis arborea* and α-bisabolol. Data are means ± standard errors (*n* = 5). Significant differences (*p* < 0.05) are indicated by letters. Lowercase letters compare the essential oils of *V. arborea* and α-bisabolol, and uppercase letters compare different concentrations.

EOVA induced a progressive increase of up to 90% and 500% in the amount of H_2_O_2_ and MDA, respectively, in the seedlings of *S. occidentalis* (Figure 5). MDA concentration increased in response to all concentrations of α-bisabolol. EOVA at 0.5% increased the activities of SOD and CAT in *S. occidentalis* seedlings (Figure 5). In contrast, the activities of SOD and CAT were reduced by α-bisabolol at 0.25 and 0.5%. SOD, CAT, and APX activities were enhanced by approximately 40, 40, and 50%, respectively, in *S. occidentalis* seedlings treated with 0.75% α-bisabolol (Figure 5).

The amount of H_2_O_2_ and MDA in *O. sativa* seedlings was significantly influenced by the treatments (Figure 5). H_2_O_2_ concentration increased by 80 and 40% in response to EOVA doses of 0.5 and 1%, respectively. Treatment with α-bisabolol at 0.25 and 0.75% increased the H_2_O_2_ concentration by approximately 50%. MDA concentration also increased in *O. sativa* seedlings in response to both EOVA and α-bisabolol at concentrations of 0.5%. Except for reduced APX, EOVA and α-bisabolol had only a minor influence on the activity of the antioxidant enzymes in *O. sativa* seedlings (Figure 5).

### 3.3 EOVA and α-Bisabolol Physiological Effects on Postemergence of *S. occidentalis* and *O. sativa*

**Figure 6:**
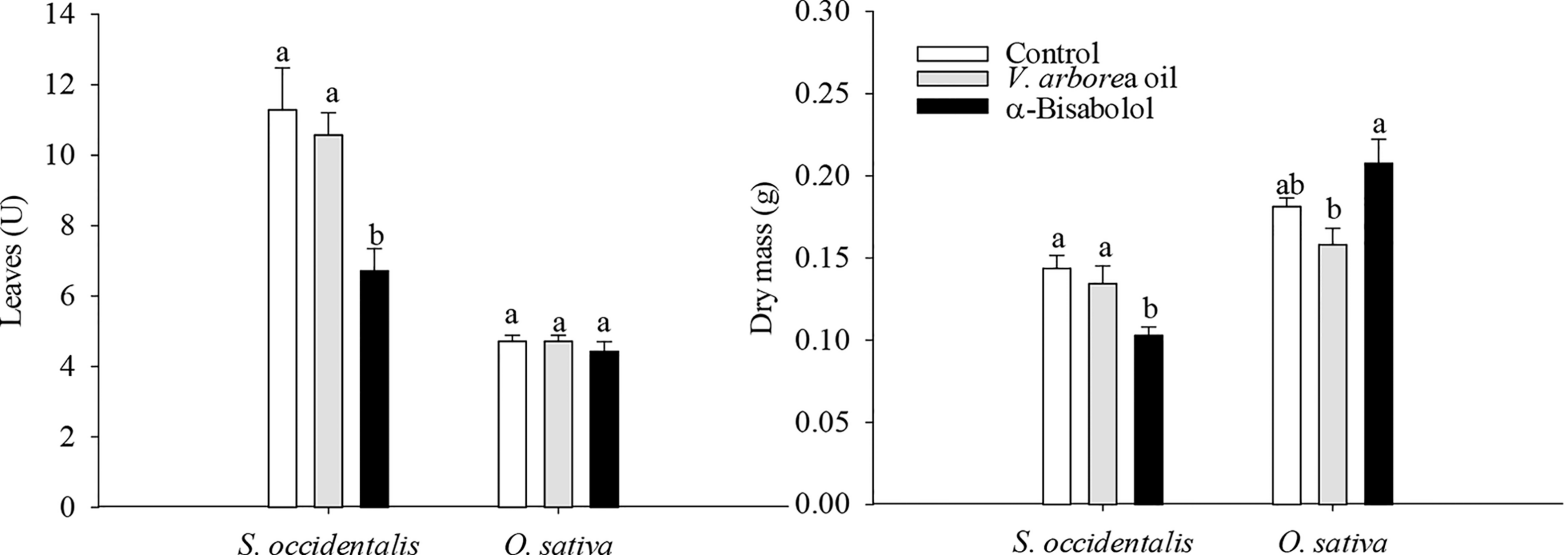
Number of leaves and shoot dry mass of plants of *Senna occidentalis* and *Oryza sativa* treated with the essential oil of *Vanillosmopsis arborea* and α-bisabolol at a 0.5% concentration. Seedlings were harvested 40 h (*S. occidentalis*) or 5 days (*O. sativa*) after treatments. Data are means ± standard errors (*n* = 7). Significant differences (Tukey’s test, *p* < 0.05) in each species are indicated by letters.

Dry mass accumulation in shoots was significantly influenced by the application of the treatments. α-Bisabolol had contrasting effects, decreasing the shoot dry mass of *S. occidentalis* by 40% while increasing the shoot dry mass of *O. sativa* by 10% (Figure 6). EOVA decreased the shoot dry mass of *O. sativa* by 15%. Moreover, the number of leaves (Figure 6) was not influenced by the treatments in *O. sativa* plants, but α-bisabolol reduced the number of leaves in *S. occidentalis* plants (Figures 6 and S5).

**Figure 7:**
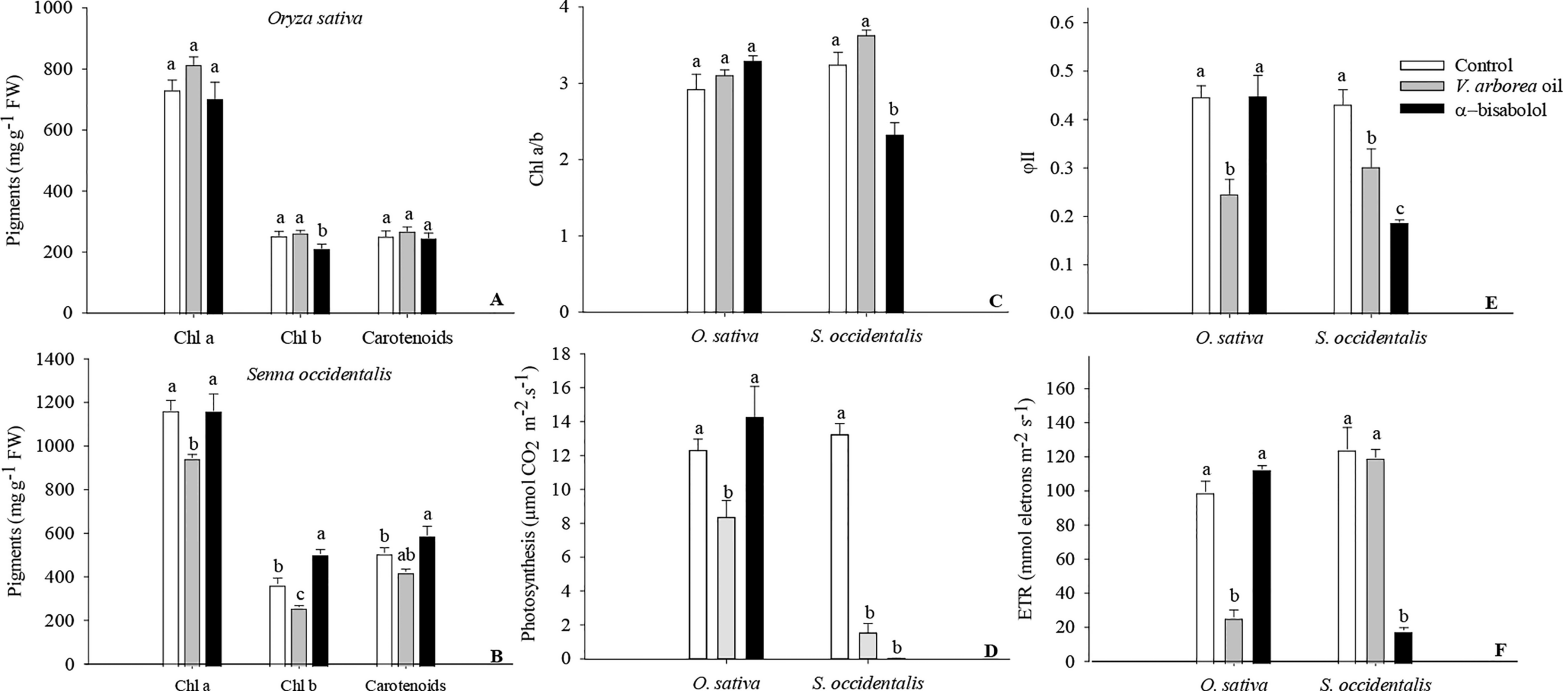
Quantification of photosynthetic pigments (A and B), chlorophyll *a*/*b* ratio (C), photosynthesis (D), photosystem II efficiency (E), and electron transport rate (F) in plants of *Oryza sativa* and *Senna occidentalis* treated with the essential oil of *Vanillosmopsis arborea* essential oil and α-bisabolol at 0.5% concentration. Seedlings were harvested 5 days (*O. sativa*) and 40 h (*S. occidentalis*) after treatments. Data are the means ± standard errors (*n* = 7). Significant differences (Tukey’s test, *p* < 0.05) in each species are indicated by letters.

The photosynthesis of the *O. sativa* and *S. occidentalis* plants was significantly influenced by the treatments (Figure 7). EOVA reduced the photosynthesis of *O. sativa* by approximately 30%, whereas the strongest effects were found for *S. occidentalis* (photosynthesis decreased by 90 and 98% in response to EOVA and α-bisabolol, respectively). EOVA also reduced the efficiency of photosystem II and the electron transport rate (ETR) in *O. sativa* plants (Figure 7). In *S. occidentalis* plants, both EOVA and α-bisabolol reduced the efficiency of photosystem II (Figure 7). In addition, α-bisabolol reduced the ETR. The amount of chlorophyll (chl) *b* was reduced by 20% in *O. sativa* plants treated with EOVA (Figure 7). However, the concentrations of all analyzed pigments decreased in response to EOVA in *S. occidentalis*, but the chlorophyll *a*/*b* ratio was not different from that of the control. Regarding *S. occidentalis*, α-bisabolol increased *chl* b and carotenoids by 40 and 15%, respectively, and decreased the chlorophyll *a*/*b* ratio by 30% (Figure 7).

**Figure 8:**
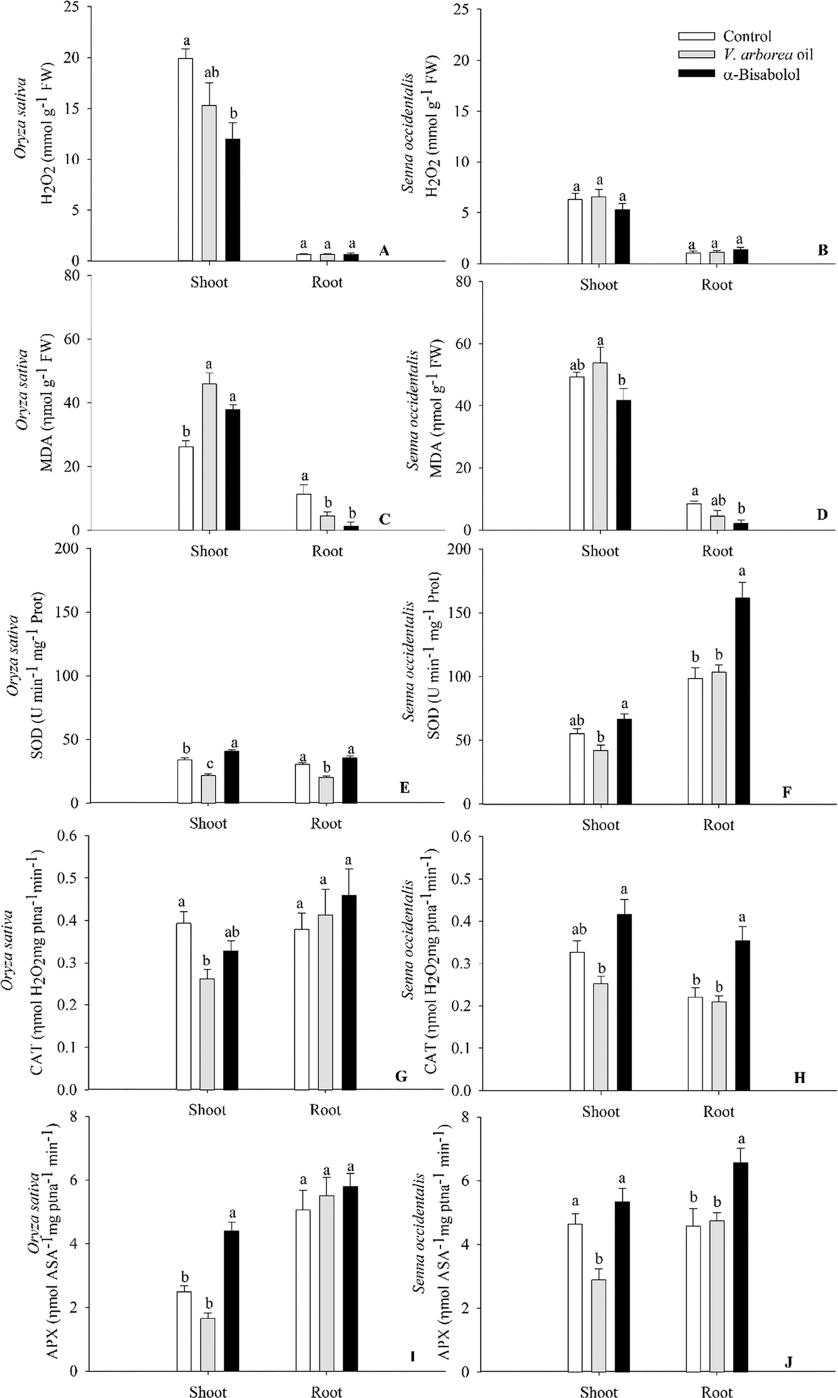
Quantification of H_2_O_2_ and malondialdehyde and enzyme activity of superoxide dismutase (SOD), catalase (CAT), and ascorbate peroxidase (APX) in plants of *Oryza sativa* (A; C; E; G: I) and *Senna occidentalis* (B; D; F; H; J) treated with the essential oil of *Vanillosmopsis arborea* and α-bisabolol at 0.5% concentration. Seedlings were harvested 5 days (*O. sativa*) and 40 h (*S. occidentalis*) after treatments. Data are the means ± standard errors (*n* = 7). Significant differences (Tukey’s test, *p* < 0.05) in each organ are indicated by letters.

The amount of H_2_O_2_ decreased in *O. sativa* shoots treated with EOVA and α-bisabolol, but MDA increased approximately 75 and 45%, respectively, in response to these treatments (Figure 8). H_2_O_2_ levels in the *S. occidentalis* plants were not significantly influenced by the treatments, but EOVA increased the amount of MDA in the shoots by 10%. MDA decreased in roots of *O. sativa* and *S. occidentalis* in response to α-bisabolol. The activities of SOD, CAT, and APX enzymes (Figure 8) decreased in *O. sativa* shoots by approximately 40, 40, and 35%, respectively, after EOVA applications, but the activities of SOD and APX increased in response to α-bisabolol. In roots of *O. sativa*, only SOD activity decreased by 35% in response to EOVA. α-bisabolol increased the activity of the SOD, CAT, and APX enzymes (Figure 8) in the roots of *S. occidentalis* plants, and EOVA decreased the activity of APX in the shoots.

## 4 Discussion

This study presents a new perspective on the use of α-bisabolol with the first description of its effects on plant metabolism and weed control. We evaluated the phytotoxic potential of the EOVA on several weed and crop species and compared the physiological mechanism of EOVA and its major component, α-bisabolol, in the pre and postemergence of the weed *S. occidentalis* and the crop *O. sativa*. The analysis of EOVA by CG-MS revealed a similar composition described by Marco et al.,^1^ except for eucalyptol. We also confirmed that the sesquiterpene α-bisabolol is the major component of this oil (Table 1), corroborating the findings of Marco et al.^1^ and Moura et al.^38^

Our results revealed that EOVA is phytotoxic to the pre-emergence of weeds, probably due to the action of its metabolites. The reduction of seed germination induced by sesquiterpenes is considered a secondary effect of several interferences in the metabolism, including inhibition of cell division, respiration, the modification of the cell membrane permeability, increase of ROS, suppression of the oxidative metabolism, and oxidative stress.^6^ In postgermination, the roots are the first seedling organ in contact with the allelochemicals, which explains their sensibility.^39^ However, the roots of the weed species *S. occidentalis* and *D. tortuosum* showed an increase in size in the lowest concentrations of followed by a reduction in the shoot size (Table 2). These results suggest that the seedlings exhibited an escape behavior from the stress induced by the allelochemicals of EOVA.

The weeds and the model species *L. sativa*, showed more severe effects concerning reduced germination and growth, and a high number of abnormal plants and inviable seeds (Figure S3). The seeds of the target species *B. pilosa*, *C. disfformis*, and *C. echinatus* were more sensitive to the application of EOVA, as seed germination was inhibited even at the lowest concentration used. Due to these different responses and levels of sensitivity (Figure S2), it was not possible to determine a pattern of response between monocots and dicots or those of the same botanical family (Asteraceae, Fabaceae, and Poaceae).

The results guided us to investigate the phytotoxic role of the major EOVA molecule α-bisabolol. For that, further experiments were focused on the weed *S. occidentalis* and crop *O. sativa*, which were the two species showing the most contrasting sensitivity to EOVA in terms of germination percentage and growth. By comparing the effects of EOVA and α-bisabolol, we noticed that EOVA was more effective at inhibiting and delaying the germination of *S. occidentalis*. This may be related to the presence of other metabolites with allelochemical potential, such as eucalyptol and eugenol, and their synergistic action with α-bisabolol.^40−43^ The reduction in α-amylase enzyme activity (Figure S4) slows starch hydrolysis and, consequently, reduces the production of energy necessary for germination and initial seedling growth,^44,45^ corroborating the germination speed index results.

In *S. occidentalis* seeds, the treatment with α-bisabolol induced a higher lipid peroxidation than EOVA, which decreased germinability. α-Bisabolol is a nonpolar sesquiterpene that can interact with membrane lipids, allowing it to enter into the cell.^46^ Once inside the cell, the α-bisabolol molecule is converted into α-bisabolol oxide A, releasing H^+^ protons and changing the electrochemical balance of the cell.^47^ It is known that membrane losses due to oxidative damage are related to increased fluidity and solute leakage.^48,49^ However, the specific effects of the conversion to α-bisabolol oxide A on cell function and electrochemical balance would depend on various factors, including concentration, exposure duration, and the type of cell being studied. Loss of viability is not always only associated with lipid peroxidation in some species.^50,51^

On the other hand, EOVA at higher concentrations induced enhanced SOD, CAT, and APX activities in *S. occidentalis* seeds, which probably decreased the damage caused by lipid peroxidation. This might be due to elevated ROS at 1% EOVA representing a high investment in defense that can compete with other functions necessary for germination. Together, these changes result in oxidative damage, that could cause cell death by activating processes of apoptosis and/or necrosis, and loss of function of essential organelles is a mechanism associated with essential oils.^48^ However, it is reasonable to suggest that the germination impairment of *S. occidentalis* seeds treated with EOVA could be related to the oxidation of other kinds of targets, such as proteins (i.e., storage proteins) and DNA. In *O. sativa* seeds, the reduction in H_2_O_2_ in response to practically all concentrations of the EOVA and α-bisabolol, and the significant increase in lipid peroxidation induced by α-bisabolol at 1% can be explained by the reduction in APX activity.

α-Bisabolol was more effective at reducing the capacity of seedlings to grow than at reducing seed germination in both species. This suggests that the changes induced by EOVAin *S. occidentalis* seedlings are related to the α-bisabolol present in the oil. *Senna occidentalis* seedlings treated with different concentrations of the α-bisabolol showed oxidative stress indicated by a much higher amount of MDA compared with the control and seedlings treated with EOVA. The seedlings of *S. occidentalis* did not quench a large amount of peroxide, which consumes ROS generated from lipid peroxidation. As the conversion of α-bisabolol into α-bisabolol oxide A produces different reactive species, such as superoxide anions (O^2–^) and hydroxyl radicals (*OH), these can also induce oxidative damage.^49^ In addition, the activity of enzymes SOD, CAT, and APX was inhibited. Further investigations are required to assess the impact of EOVA and α-bisabolol on ROS-producing organelles like mitochondria to elucidate the specific effects on respiratory processes. Oxidative stress causes cell death and increases systemic necrosis in various organs, eventually spreading throughout the entire plant (Figure 4), reducing growth and causing plant death.^11,49^ Thus, seedlings from seeds treated with α-bisabolol would not be able to recover. The antioxidant system was suppressed in *O. sativa* seedlings, consequently, lipid peroxidation increased, which reduced the height of the seedlings. However, this lipid peroxidation was not as severe as in *S. occidentalis*, providing a greater chance of recovery for *O. sativa* seedlings.

This oxidative stress induced by the application of α-bisabolol in weed seedlings suggests a high activity of this molecule in postemergency. Therefore, 0.5% of EOVA and α-bisabolol were applied to the seedlings of *S. occidentalis* and *O. sativa* evaluated at the postemergence stage. Treatments induced greater changes in the physiology of the weed species *S. occidentalis* than in the crop *O. sativa*; consequently, with a faster deterioration in the former. The changes in the photosynthesis of the *O. sativa* plants were driven in response to the synergistic action of all metabolites present in the EOVA since the use of α-bisabolol did not influence this process.^54^ The reduced electron transport in *O. sativa* plants may be related to the inhibition of quinone oxidation and the primary acceptor of electrons, which blocks the electron transport chain (ETC), thus inducing changes in photosystem II^10^ and decreasing photosynthesis.^55^ However, these modifications were insufficient to drastically reduce the photosynthesis of these plants, and the dry mass of *O. sativa* plants was not affected during the evaluated time.

Regarding *S. occidentalis* plants, EOVA and α-bisabolol reduced carbon fixation but with some differences in the process. Treatment with EOVA decreased chlorophyll *a* and *b* contents, which is a common response to allelochemicals and can occur as a result of inhibition of chlorophyll synthesis or induction of chlorophyll degradation.^56^ This reduction can influence the harvesting of light energy and electron transfer in the reaction centers, inhibiting the activity of enzymes and thus decreasing ATP synthesis.^8^ Together, these changes could favor an accumulation of unfixed CO_2_ that disrupts stomata, ultimately resulting in decreased photosynthesis.^57^ However, until the time of sampling, these modifications were insufficient to reduce the number of leaves and the dry mass of the *S. occidentalis* plants.

In contrast, the reduction of photosynthesis in *S. occidentalis* plants treated with α-bisabolol is due to the inhibition of the electron transport rate, which reduces the efficiency of photosystem II and decreases ATP. These plants showed higher chlorophyll *a* and *b* contents and consequent reduction in the chl *a*/*b* ratio. Two antenna systems exist in photosystems: the internal antenna complex, which does not contain chl *b*, and the light-harvesting complex, which contains chl *b*. Thus, the lower chl *a*/*b* ratio may be related to the degradation of internal antenna complexes, as they are more sensitive than the light-harvesting complexes.^58^ α-Bisabolol induced the drying of leaves of *S. occidentalis* plants, causing them to fall and consequently reducing the dry mass of the plants.

The increase in lipid peroxidation in the shoots of the *O. sativa* plants treated with EOVA suggests that the H_2_O_2_ produced leads to oxidative damage, inhibiting the activity of the enzymes SOD, CAT, and APX.^54^ The application of α-bisabolol on *O. sativa* plants may have induced depolarization of the cell membrane, increasing its permeability and lipid peroxidation, thus causing leakage of cell contents and, consequently, cell death.^59,49^ The increase in lipid peroxidation in the shoots of *S. occidentalis* plants treated with EOVA is due to the inhibition of the activity of antioxidant enzymes associated with changes in photosynthesis.^6^ The lower lipid peroxidation in plants treated with α-bisabolol is due to increased carotenoids, antioxidant pigments, and the activity of the enzymes SOD and CAT.^49^ In this way, EOVA and α-bisabolol can influence physiological processes involved in the growth of these plant species.

Our experiments showed that EOVA has phytotoxic potential because it reduced the growth and development of several target species. The mode of action of EOVA involves inhibiting the activity of antioxidant enzymes and changing the electrochemical balance of the cell as well as increasing lipid peroxidation. The inhibitory effects varied in severity depending on the developmental stage of the target plant. In postemergence, EOVA and α-bisabolol changed the photosynthetic system, pigment concentrations, and antioxidant system, but *O. sativa* showed low sensitivity to their action. Moreover, the rapid deterioration of *S. occidentalis* plants treated with α-bisabolol indicates that this molecule could be classified as a contact molecule. Our results suggested a selective action of α-bisabolol and EOVA as a bioherbicide, which is very promising for weed control.

## Supporting Information Available

Data from the statistical analysis of Experiment 1, with equations, *p* value, coefficient of variation, and determination coefficient; seeds and seedlings abnormals, chlorotic, and necrotic of different target species submitted to 1% of the essential oil of *Vanillosmopsis arborea*; data on germination percentage, germination speed index, shoot length, and root length were used for grouping analysis of species treated with the essential oil of *Vanillosmopsis arborea*; seeds mortality after submission to different concentrations of essential oil of *Vanillosmopsis arborea*; data from the statistical analysis of Experiment 2, with equations, p.value, coefficient of variation, and determination coefficient; α-amylase activity in *Senna occidentalis* (A) and *Oryza sativa* (B) seeds in response to different concentrations of essential oil of *Vanillosmopsis arborea* and α-bisabolol; and morphology of *Oryza sativa* and *Senna occidentalis* of plants subjected to spray applications of *Vanillosmopsis arborea* essential oil and α-bisabolol at 0.5% concentration (PDF)
