# Supplementary material for: New Perspective on the Use of α-Bisabolol for Weed Control
Source: J Agric Food Chem. 2024 Mar 19;72(12):6289–301. doi: 10.1021/acs.jafc.3c08566 (PMC11197090; doi:10.1021/acs.jafc.3c08566)

Supporting Information

A new perspective on the use of α-bisabolol for weed control

Josyelem Tiburtino Leite Chaves [a], Geovane da Silva Dias [a], Marina Mariá Pereira [a], Ludmila da Silva Bastos [a], Maria Isabel Almeida Souza [a], Larissa Fonseca Andrade Vieira [b], Ana Cardoso Clemente Filha Ferreira de Paula [c], Cláudia Araújo Marco [d], Paulo Eduardo Ribeiro Marchiori [a], Elisa Monteze Bicalho [a*]

[a] Laboratório de Crescimento e Desenvolvimento de Plantas, Setor de Fisiologia Vegetal, Universidade Federal de Lavras, CEP 37200-000, Lavras, Minas Gerais, Brasil.

[b] Laboratório de Citogenética, Universidade Federal de Lavras, CEP 37200-000, Lavras, Minas Gerais, Brasil.

[c] Instituto Federal de Minas Gerais, CEP 38900-000, Bambuí, Minas Gerais, Brasil.

[d] Laboratório Interdisciplinar em Produtos Naturais, Centro de Ciências Agrárias e da Terra, Universidade Federal do Cariri, CEP 63130-025, Crato, Ceará, Brasil.

*Corresponding author: E-mail addresses: elisa.bicalho@ufla.br

Table S1. Data from the statistical analysis of Experiment 1

	Germination percentage	Germination Speed Index	Stem	Root	Mortality
L. sativa	f= 96.32-116.87x+37.29x² p.value= 0.0028 R^2= 0.97 CV = 10.3%	f= 21.78-44.04x+24.70x² p.value= 0.0034 R^2= 0.96 CV= 18.24%	f= 0.99-1.84x+0.95x² p.value= 0.001 R^2= 0.78 CV= 13.79%	f= 0.79-1.35x+0.62x² p.value= 0.0005 R^2= 0.89 CV= 20.35%	f=2.804+118.7x-42.11x² p.value=0.009 R^2= 0.98 CV= 14.13
O. sativa	CV= 4.08 % Mean= 92.13	f= 21.55exp(-1.64x) p.value= 0.0001 R^2= 0.69 CV= 7.45%	f= 3.09-5.94x+3.88x² p.value= 0.0001 R^2= 0.87 CV= 20.57%	f= 4.99-12.55x+8.43x² p.value= 0.00001 R^2= 0.79 CV= 19.91%	CV= 37.79 Mean= 7.9
S. occidentalis	f= 99.5181-4.9163x-53.84x² p.value= 0.0227 R^2= 0.90 CV= 14.4%	f= 21.31-30.67x+13.80x² p.value= 0.041 R^2= 0.82 CV= 27.36%	f= 3.73exp(-2.34x) p.value= 0.001 R^2= 0.84 CV= 22.07%	f= 1.81-1.71x+0.13x² p.value= 0.08 R^2= 0.50 CV= 31.95%	f=0.3062+7.357x+50.17x² p.value=0.025 R^2= 0.95 CV= 55.71
D. tortuosum	f= 96.07exp(-0.36x) p.value= 0.0001 R^2= 0.82 CV= 8.95%	f= 11.68/ (1+exp (-(x-1.07) /-0.39)) p.value= 0.0001 R^2= 0.68 CV= 13.36%	f= 1.50exp(-1.85x) p.value= 0.001 R^2= 0.85 CV= 14.54%	f= 1.87exp(-1.45x) p.value= 0.003 R^2= 0.72 CV= 31.95%	f=3.811+35.939x-6.458x² p.value=0.026 R^2= 0.95 CV= 42.08
C. echinatus	f= 84.54exp(-14.60x) p.value= 0.0001 R^2= 0.93 CV= 36.7%	f= 12.05exp(-23.32x) p.value= 0.0001 R^2= 0.92 CV= 34.89%	f=4.85exp(-19.37x) p.value= 0.0001 R^2= 0.90 CV= 38.91%	f=5.65exp(-20.76x) p.value= 0.0001 R^2= 0.91 CV= 41.23%	f= 91.61/ (1+exp (-(x-0.0550)/0.03)) p.value=0.0001 R^2= 0.90 CV= 9.48
C. disfformis	f= 74.39exp(-24.29x) p.value= 0.0069 R^2= 0.90 CV= 32.76	f= 3.48exp(-21.22x) p.value= 0.001 R^2= 0.91 CV= 33.57	-	-	-
B. pilosa	-	-	-	-	f= 92.17/ (1+exp (-(x-0.069)/0.03)) p.value=0.0001 R^2= 0.89 CV= 14.4

Figure S1: Seeds and seedlings of different target species submitted to 1% of the essential oil of *Vanillosmopsis arborea*. Bars = 0.5cm.

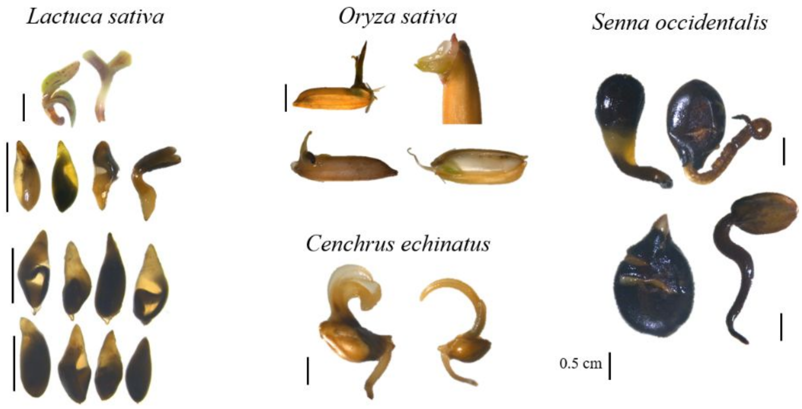

Figure S2: Grouping analysis of target (weed) and nontarget crop species treated with the essential oil of *Vanillosmopsis arborea*. and based on the variables Data on germination percentage, germination speed index, shoot length, and root length were used for this analysis.

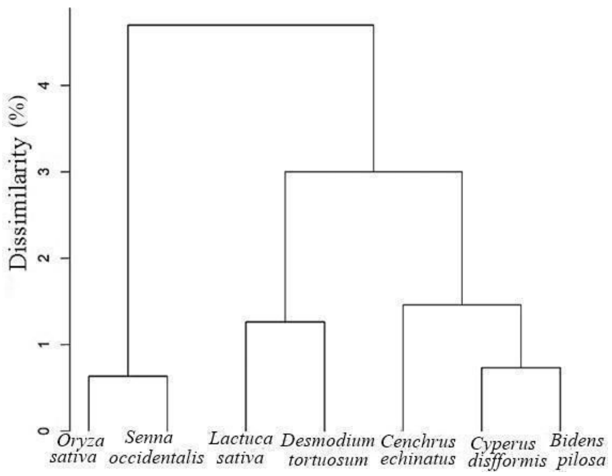

Figure S3: Seeds mortality after submission to different concentrations of essential oil of *Vanillosmopsis arborea.*

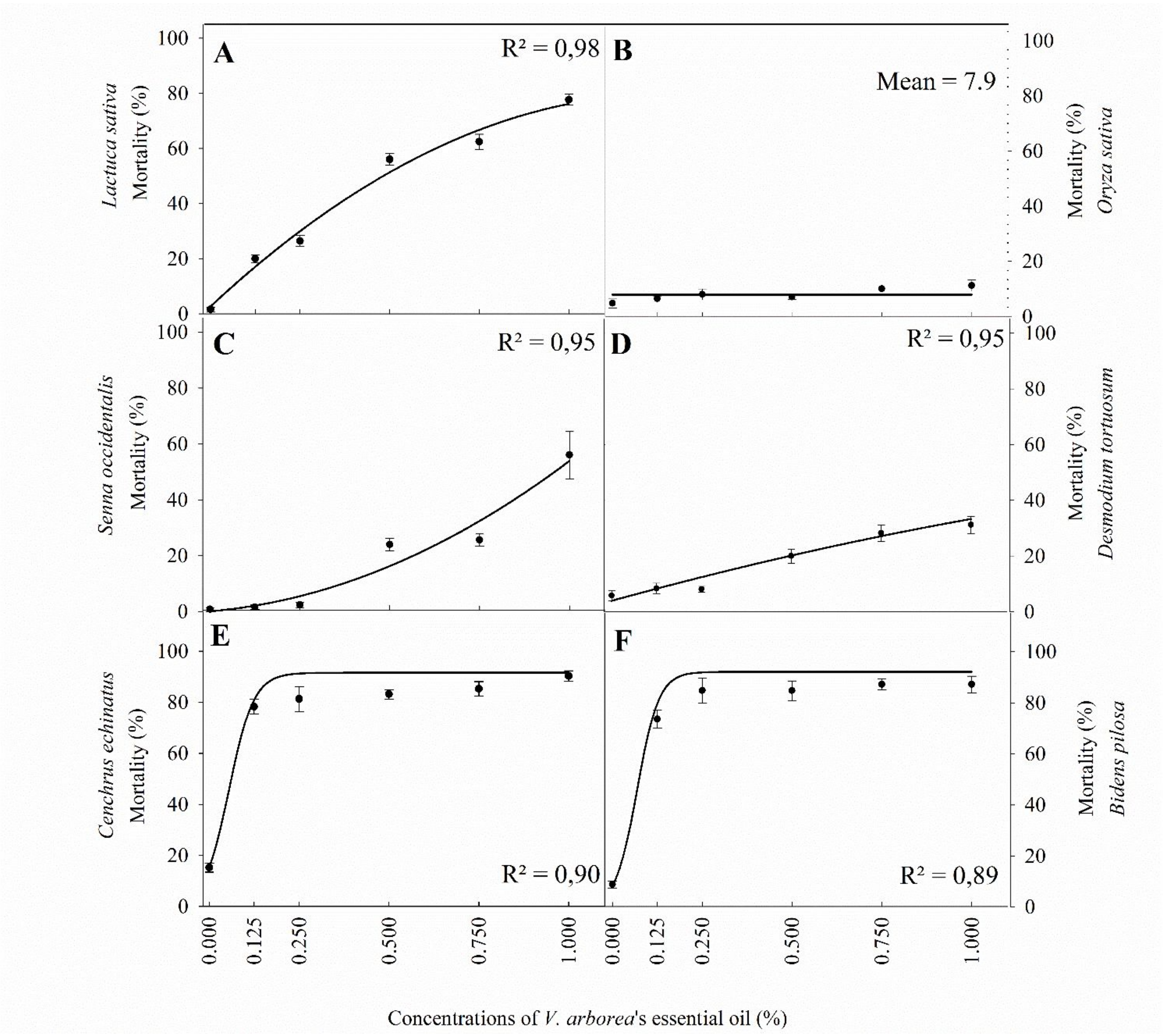

Table S2: Data of the statistical analysis of germination test in Experiment 2

<table>
<tr><th></th><th>Germination percentage</th><th>Germination Speed Index</th><th>Shoot</th><th>Root</th></tr>
<tr><td>Oryza sativa
Essential oil of V. arborea</td><td rowspan="2">CV= 6.54 %
Mean= 91.52</td><td rowspan="2">f= 11.27-6.719x+4.00x²
p.value= 0.0002
R²= 0.97
CV= 8.19%</td><td rowspan="2">f= 2.41-1.98x+0.748x²
p.value= 0.1968
R²= 0.947
CV= 24.69%</td><td rowspan="2">f=5.138-3.23x+0.933x²
p.value= 0.4114
R²= 0.94
CV= 21.49%</td></tr>
<tr><td>Oryza sativa
α-bisabolol</td></tr>
<tr><td>Senna occidentalis
Essential oil of V. arborea</td><td>f= 96.09-12.57x-33.82x²
p.value= 0.037
R²= 0.99
CV= 9.8%</td><td>f= 21.10-31.72x+17.63x²
p.value= 0.001
R²= 0.87
CV= 13.28%</td><td rowspan="2">f= 4.81-10.33x+7.26x²
p.value= 0.001
R²= 0.89
CV= 15.09%</td><td rowspan="2">f= 3-2.03x+3.5x²
p.value= 0.002
R²= 0.7
CV= 23.08%</td></tr>
<tr><td>Senna occidentalis
α-bisabolol</td><td>CV= 9.8 %
Mean= 90.72</td><td>f= 21.15-0.28x+3.69x²
p.value= 0.36
R²= 0.53
CV= 13.28%</td></tr>
</table>

Figure S4: α-Amylase activity in *Senna occidentalis* (A) and *Oryza sativa* (B) seeds in response to different concentrations of essential oil of *Vanillosmopsis arborea* and α-bisabolol. * The lowercase letters compare *V. arborea* essential oil and α-bisabolol. The uppercase letters compare concentrations of oil or α-bisabolol.

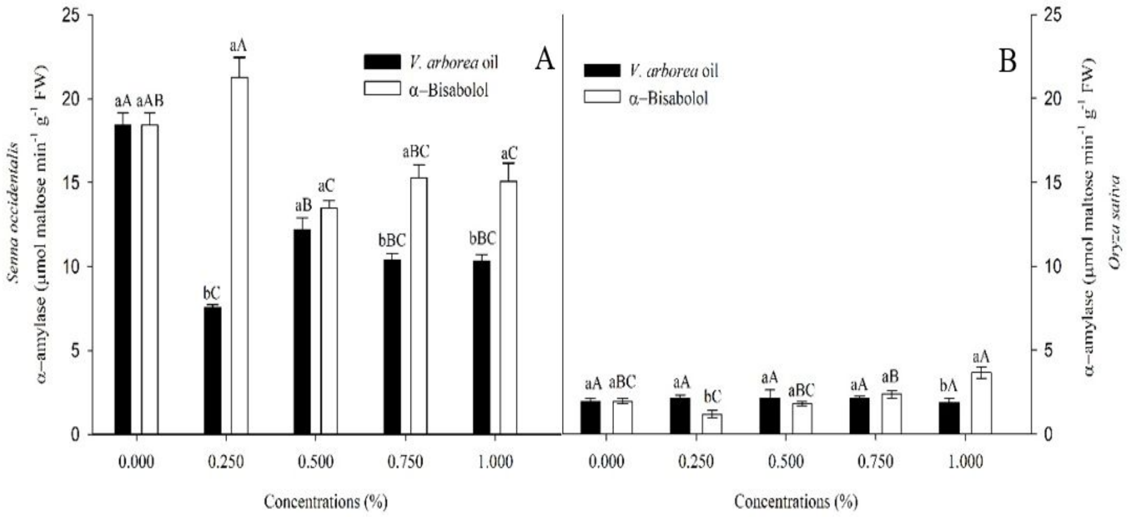

Figure S5: Morphology of *Oryza sativa* (A) and *Senna occidentalis* (B) of plants subjected to spray applications of *Vanillosmopsis arborea* essential oil and α-bisabolol at 0.5% concentration. Five days and 40 hours after treatments for *Oryza sativa* and *Senna occidentalis*, respectively. Bar= 1 cm

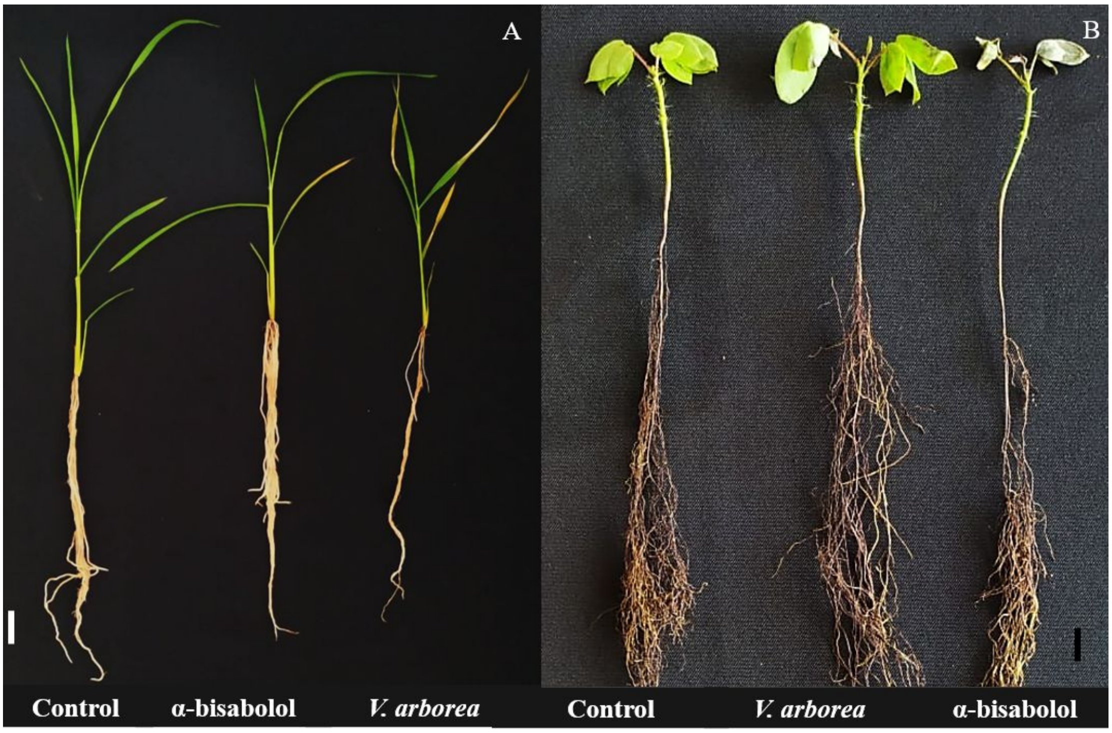

Supplement: Supplementary file 1 — jf3c08566_si_001.pdf [file jf3c08566_si_001.pdf]
